# The Center for Disease Control and Prevention’s (CDC) Youth Violence Prevention Centers: Paving the Way to Prevention

**DOI:** 10.1007/s10935-016-0433-8

**Published:** 2016-03-30

**Authors:** James A. Mercy, Alana M. Vivolo-Kantor

**Affiliations:** Division of Violence Prevention, National Center for Injury Prevention and Control, Centers for Disease Control and Prevention, 4770 Buford Highway MSF64, Atlanta, GA 30341 USA

When you think about violence in the United States in recent years, it is clear that we have been transfixed by a seemingly unending series of tragedies associated with mass shootings. While those events deserve our utmost attention, what has been largely ignored is the fact that we lose an average of 12 youth 10–24 years of age to homicide each day in this country [Centers for Disease Control and Prevention (CDC), 2014]. In effect, every day in the United States we lose more young people to “unrelated” homicides than occur in a typical mass shooting. The health burden of these “unrelated” homicides and associated nonfatal violence dwarfs that of the mass shootings of which we, as a society, have been so acutely aware. All the while, in the dark, the broader and much larger problem of youth violence continues, largely unacknowledged and unaddressed. However, despite the relative obscurity this issue has faced, great progress is being made in understanding how communities can work together to prevent it.

## Youth Violence Is an Urgent Public Health Problem

By any measure, and relative to other health issues that garner far greater attention and resources, youth violence is an urgent public health problem. Overall, homicide is the 3rd leading cause of death among 10–24 year olds in the United States (CDC, 2014). What is even more disturbing is that homicide has been the leading cause of death for 10- to 24-year-old African American males and females for decades (CDC, 2014). In addition to these deaths are nonfatal forms of youth violence that also have substantial health consequences. In 2013, over 1500 youth 10–24 years of age were estimated to have been treated in hospital emergency departments each day as a result of injuries associated with assaults (CDC, 2014).

The consequences of youths’ exposure to violence, as victims or witnesses, extend far beyond physical injury and have the potential for deep and lasting impacts on their well-being throughout their entire lives (Hillis, Mercy, & Saul, 2016). These potentially long-lasting consequences include increases in the risks of sexually transmitted infections (including HIV), mental health problems, reproductive health problems, and non-communicable diseases, including heart disease, cancer, and diabetes (Fowler, Tompsett, Braciszewski, Jacques-Tiura, & Baltes, 2009; Hillis et al. 2016). Studies addressing the biologic underpinnings of such consequences demonstrate that violence-associated toxic stress may cause damage to the nervous, endocrine, circulatory, reproductive, respiratory, and immune systems (National Scientific Council on the Developing Child, 2005). We also understand that youth violence has very serious consequences for communities as well in that it creates fear and mistrust, restricts freedom of movement, and inhibits economic development (Morrel-Samuels, Bacallao, Brown, Bower, & Zimmerman, 2016).

## Youth Violence Is Preventable

We actually know a lot about how to prevent youth violence. There is now a wealth of scientific evidence supporting strategies that can be implemented with individuals, in schools, and within communities to effectively reduce and prevent youth violence. The following nine overlapping strategies have been shown to directly impact youth violence: (1) building children’s and adolescents’ skills and competencies to choose nonviolent, safe behaviors; (2) fostering safe, stable, nurturing relationships between young people and their parents and caregivers; (3) building and maintaining positive relationships between young people and caring adults in their community; (4) developing and implementing school-wide activities and policies to foster social connectedness and a positive environment; (5) improving and sustaining a safe physical environment in communities and creating spaces to strengthen social relationships; (6) building viable and stable communities by promoting economic opportunities and growth; (7) facilitating the social cohesion and collective efficacy of the community; (8) changing societal norms about the acceptability of violence and willingness to intervene; and (9) changing the social and structural conditions that affect youth violence and lead to health inequity (David-Ferdon & Simon, 2014).

## Youth Violence Prevention Centers (YVPCs): Working With Communities to Apply and Measure What Works

It is clear that youth violence is an urgent problem and that we actually know a lot about how to prevent it. The challenge that has been evident for many years, however, is how to help communities take this knowledge and effectively apply it to their own situations and contexts. CDC, through the Youth Violence Prevention Centers (YVPCs), has been investing in meeting this challenge for over 15 years (Matjasko, Massetti, & Bacon, 2016). Through collaborative partnerships and research in high-risk communities across the country, YVPCs have established the fundamentals of successful ways that communities can come together to prevent youth violence and measure their impact. The YVPCs were charged with implementing and evaluating multifaceted, comprehensive, and evidence-based primary prevention approaches to reduce youth violence. Thus, the YVPCs are unique in that they are building the evidence around implementing a comprehensive youth violence prevention package. However, identifying and implementing packages of evidence-based programs that reach multiple levels of the social ecology is both an art and a science. This special issue highlights many of the key lessons that have been learned about the art and science of preventing youth violence.

One of the most basic lessons learned is the importance of considering differences across communities, because the types of evidence-based interventions appropriate for one community may be less relevant for another. Kingston, Bacallao, Smokowski, Sullivan, and Sutherland (2016) review how different YVPCs have approached this issue and provide detail on the importance of not only applying the best scientific evidence, but also matching the best available evidence with a community’s strengths and needs. The importance of both the capacity and readiness of a community to adopt and implement these packages of evidence-based programs cannot be emphasized enough.

Another key area where the YVPCs have learned valuable lessons is how academic institutions can best engage and partner with high-risk communities. Morrel-Samuels and colleagues (2016) provide examples from the YVPCs that demonstrate how key community engagement and partnerships are to building the necessary infrastructure and capacity to do this work. Academic institutions play a very valuable role in assisting communities in youth violence prevention activities. The establishment of trust between academic institutions and communities across multiple sectors is an essential prerequisite to preventing violence at the community level (Massetti & Vivolo, 2010). In addition, transparency in communication and action lays the foundation for successful implementation. These trusting and transparent relationships between academic and community partners assist in the success of sustainability efforts. For example, the University of Colorado Boulder Youth Violence Prevention Center and the University of North Carolina Academic Center for Excellence in Youth Violence Prevention at the University of North Carolina, Chapel Hill have worked with their respective communities to set up non-profit organizations that are tasked with continuing the implementation of evidence-based programs and securing funding from multiple sources long after federal funding ends.

YVPC’s have also provided critical information about how to measure youth violence and how to implement study designs that can effectively evaluate the impact these multifaceted programs are having in the community. The YVPCs have identified and used a variety of data sources (e.g., crime and emergency department data) to monitor the magnitude of youth violence as well as violence trends in communities over extended periods of time (Masho, Bishop, & Farrell, 2016). Evaluating the effectiveness of multiple strategies aimed at preventing violence at the community level is no easy feat. The YVPCs have been creative in applying the most appropriate methods for assessing impact in an entire community. Randomized controlled trials, the gold standard in evaluation, may not be feasible in this context and may be too costly for the evaluation of community-wide impact (Farrell, Henry, Bradshaw, & Reischl, 2016).

With the current round of YVPCs coming to a close, promising results are emerging. For example, the Michigan Youth Violence Prevention Center (MiYVPC) has partnered with universities, economic development organizations, health departments, hospitals, police departments and community-based organizations to implement and evaluate several complementary strategies to prevent youth violence. As a result of MiYVPC’s combined collaborative efforts in Flint, MI over 5 years, youth were 5 % less likely to be victims of violent assault and experienced a 38 % decrease in assault-related injuries compared to youth in other, comparable areas (Heinze et al., 2016). Also, the Virginia Commonwealth University’s Clark-Hill Institute for Positive Youth Development YVPC empowers youth, schools, families, and other stakeholders to promote the healthy, safe, and positive development of youth through evidence-based practices. In one community that received a three-year comprehensive school-based intervention, the Clark-Hill Institute and their partners were able to achieve a 100 % reduction in the rate of ambulance pickups for violence-related injuries among youth (Masho et al., 2016).

## Moving Forward by First Looking Back

Over time, the YVPCs have helped to provide much needed answers to key questions in the youth violence prevention literature. Moreover, the lessons learned from the current round of YVPCs are also helping to inform the next generation of YVPCs. As described in this special issue, the programs selected for implementation by YVPCs and their partners showcase some of the best evidence in individual-, school-, and family-based prevention programming. However, little evidence exists of the effectiveness of community- or policy-level strategies. Thus, in September 2015, CDC funded three YVPCs (and will fund two additional centers in September 2016) to build on the evidence gleaned by the last round in order to not only implement and evaluate community- and policy-level interventions to achieve community-wide impact, but also to assess and enhance community capacity and readiness to embark on this work. Our continued efforts in supporting the YVPCs to reach their goals and objectives will enable our partners to successfully prevent and reduce youth violence across communities.

## Conclusion

The YVPCs are contributing mightily to advancing the practice of youth violence prevention. They are using science to determine inform how communities can be successful in using the best available evidence for prevention. They are shedding much needed light on an issue that demands urgent attention. Given the lessons learned from this important investment, there is every reason to believe that our understanding and capacity to prevent youth violence will make a difference. The lessons learned during the relative short tenure of the YVPCs are consistent with the lessons from the public health community’s much longer experience with the prevention of infectious and chronic diseases. Youth violence can be prevented if communities, their academic and government institutions, and their citizens start now, act wisely, and work together.
